# Novel Foxp3^−^ IL-10^−^ Regulatory T-cells Induced by B-Cells Alleviate Intestinal Inflammation in Vivo

**DOI:** 10.1038/srep32415

**Published:** 2016-09-01

**Authors:** Tzu-Yu Shao, Ling-Hui Hsu, Chien-Hui Chien, Bor-Luen Chiang

**Affiliations:** 1 Graduate Institute of Clinical Medicine, College of Medicine, National Taiwan University, No. 1, Chang-Te Street, Taipei 10048, Taiwan; 2 Graduate Institute of Immunology, College of Medicine, National Taiwan University, No. 1, Chang-Te Street, Taipei 10048, Taiwan; 3 Department of Medical Research, National Taiwan University Hospital, No. 7 Chung-Shan South Road, Taipei 10002, Taiwan

## Abstract

Recent studies have revealed various Foxp3^−^ regulatory T (Treg) cell subsets effectively protect mice from colitis. In the present study, we demonstrated that B cells induced a particular subset of regulatory T (Treg-of-B) cells, expressing programmed cell death 1 (PD-1), inducible costimulator (ICOS), lymphocyte-activation gene 3 (LAG3), glucocorticoid-induced tumor necrosis factor receptor (GITR), and OX-40, did not express Foxp3. Treg-of-B cells produced abundant levels of IL-10 and low levels of IL-4 and TGF-β. Adoptive transfer of Treg-of-B cells protected mice from CD4^+^CD45RB^hi^ T-cell-induced colitis, including infiltration of leukocytes, depletion of goblet cells, epithelial hyperplasia, and inhibition of Th1 and Th17 cytokines. These features were similar to IL-10-producing type 1 regulatory T (Tr1) cells; however, IL-10-deficient Treg-of-B cells maintained their suppressive function *in vitro* as well as *in vivo*, while the regulation of Tr1 cells depended on IL-10. In conclusion, Treg-of-B cells protected against experimental colitis through an IL-10-independent mechanism. We reported a novel subpopulation of regulatory T cells was different from conventional Foxp3^+^ Treg and IL-10-producing Tr1 cells.

Regulatory T (Treg) cells play an important role in controlling the immune response and inducing tolerance. Over the years, the transcription factor of forkhead box P3 (Foxp3) has been considered as a critical marker of regulatory T cells, and essential for the development and maintenance of thymus-derived Treg cells and peripheral-derived Treg cells^1^. However, several Foxp3^−^ suppressive T cell subsets have identified in intestines and functionally controlled peripheral immunity^2^. For example, type 1 regulatory T (Tr1) cells, a memory T lymphocyte subset expressing CD49b and lymphocyte-activation gene 3 (LAG3), produce interleukin (IL)-10 to suppress T cell response^3^. In addition, CD4^+^CD25^−^LAG3^+^ T cells produce IL-10 to maintain peripheral immune status^4^,^5^.

Recent studies have shown that naïve B cells can promote the generation of Foxp3^−^ regulatory T cells, referred to here as Treg-of-B cells^6^. Treg-of-B cells could suppress the rejection of allogenic heart transplants and also alleviate asthma symptoms^6^,^7^. We demonstrated that splenic B cells and peritoneal B-1a cells as well as Peyer’s patch B cells induced Treg-of-B cells that have many Treg-associated features, such as the production of IL-10 and the expression of CD25, cytotoxic T lymphocyte antigen 4 (CTLA4), inducible costimulator (ICOS), OX40 (CD134), programmed death-1 (PD-1), glucocorticoid-induced tumor necrosis factor receptor (GITR), and LAG3^7^,^8^,^9^,^27^. Although the characteristics of Treg-of-B cells was very similar to Tr1 cells, Treg-of-B cells did not express CD49b or CD103, two integrin proteins commonly detected in Tr1 cells^8^. Moreover, compared with Tr1 cells, both the development and function of Treg-of-B cells were IL-10-independent^9^. Therefore, the present study investigated further the effect of Treg-of-B cell-based therapy using IL-10-deficient Treg-of-B cells both *in vitro* and *in vivo*.

Inflammatory bowel diseases (IBD) are chronic inflammatory diseases, causing by innate and adaptive immune disorder in intestine and colon^10^,^11^,^12^. Because of the potential for maintaining gut immune homeostasis, Treg cells represent a promising strategy to treat IBD^13^. Many studies have shown that the transfer of Foxp3^+^ Treg cells, populations enriched in CD45RB^lo^, can attenuate or prevent the development of colitis, both by secreting IL-10 and TGF-β and by preventing the activation and function of effector T cells^13^,^14^,^15^. Also, Foxp3^−^ Treg cell subsets, Tr1 and CD4^+^CD25^−^LAG3^+^ T cells, could suppress immune responses and prevent colitis^4^,^16^. Furthermore, B cells could restore and promote the expansion of Treg cells to suppress the development of experimental colitis^17^,^18^.

In this study, we investigated the role of IL-10 in the regulatory mechanism of Treg-of-B cells in a murine model of IBD. Treg-of-B cells, induced by anti-CD3 and anti-CD28 antibodies in the presence of B cells, suppressed the proliferation of T cells in an IL-10-independent manner. Adoptive transfer of Treg-of-B cells protected mice from T cell-mediated experimental colitis. Although Treg-of-B cells expressed LAG3 and IL-10 as IL-10-producing Tr1 cells did, we showed that IL-10-deficient Treg-of-B cells exerted suppressive ability *in vitro* as well as *in vivo*. The results indicated that Treg-of-B cells possessed IL-10-independent regulatory function and differed from Foxp3^+^ nTreg and IL-10-producing Tr1 cells.

## Result

### The characteristics of Treg-of-B cells

**Figure 1:**
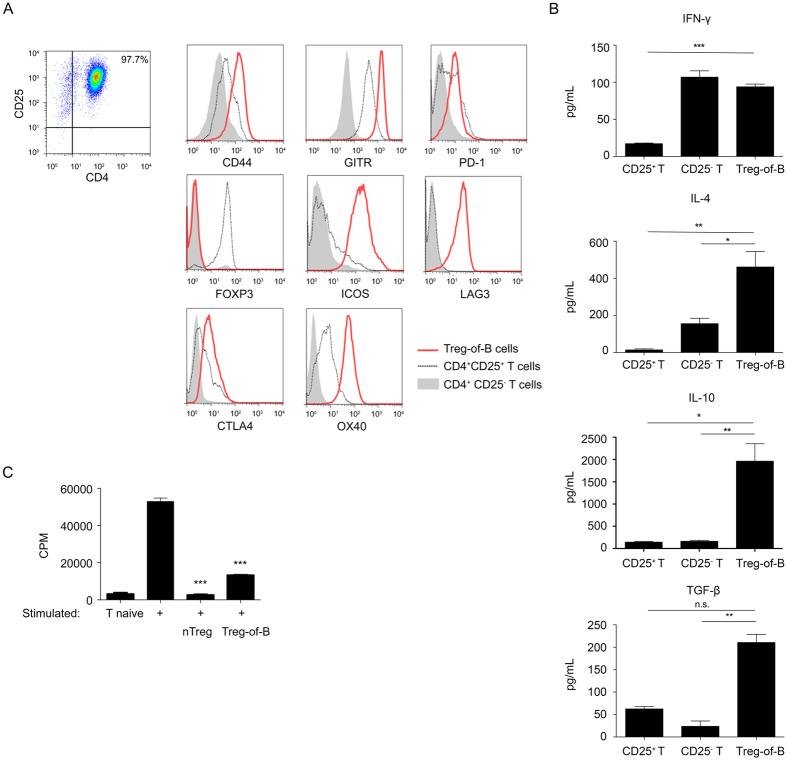
The characteristics of Treg-of-B cells. (**A**) The histograms represent the expression of each marker on gated CD4^+^CD25^+^ Treg-of-B cells, CD4^+^CD25^+^ nTreg cells or CD4^+^CD25^−^ naïve T cells.(**B**) The cytokine levels of stimulated Treg-of-B cells, nTreg cells, and naïve T cells were analyzed by ELISA. (**C**) The suppressive function of Treg-of-B cells was analyzed with nTreg cells as a suppressor control. *P < 0.05; **P < 0.01; ***P < 0.001; not significant (NS) indicates P > 0.05. The results are represented as the mean ± SEM. Significant differences were calculated using Student’s *t*-test. The data are representative of three independent experiments.

To analyze the characteristics, Treg-of-B cells were compared to both naïve T cells and natural regulatory T (nTreg) cells. We found that Treg-of-B cells expressed high levels of Treg-related molecules, such as CD44, GITR, ICOS, LAG3, and OX40. Treg-of-B cells also expressed low levels of PD-1 and CTLA4. Unlike nTreg cells, Treg-of-B cells did not express Foxp3 (Fig. 1A). We next examined the cytokine profile of Treg-of-B cells. Treg-of-B cells produced higher levels of IL-10, IL-4, and transforming growth factor (TGF)-β compared to that of naïve T cells. Treg-of-B cells also produced higher level of interferon (IFN)-γ than that of nTreg cells (Fig. 1B).

To examine the suppressive ability of Treg-of-B cells, a suppression assay was performed. Treg-of-B cells successfully inhibited effector T cell proliferation (Fig. 1C). Despite the absence of Foxp3 expression, the suppressive capacity of Treg-of-B cells was similar to nTreg cells. Together, these data suggested that B cells induced functional Foxp3^−^ regulatory T cells that produce IL-10.

### Adoptive transfer of Treg-of-B cells protects against colitis

**Figure 2:**
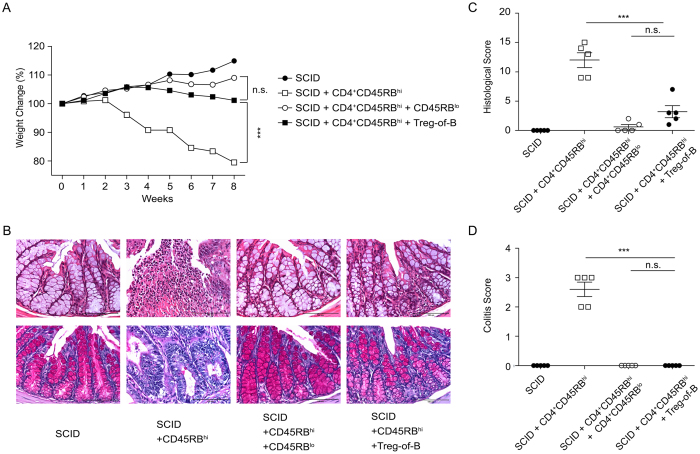
Adoptive transfer of Treg-of-B cells protected mice against colitis. (**A**) Relative body weight changed over time, (**B**) histological sections of the colon (*top*, H&E staining; *bottom*, periodic acid-Schiff staining; scale bar, 100 μm) and (**C**) histological scores of the colon sections are shown. (**D**) Disease severity scoring of mice with colitis was carried out eight weeks after the transfer of CD4^+^CD45RB^hi^ T cells. The results are shown as the mean ± SEM. Significant differences were calculated using a one-way ANOVA. ***P < 0.001; not significant (NS) indicates P > 0.05 for select comparisons. The statistics are shown in the last time in (**A**). There were 5 mice per group. The data are representative of two independent experiments.

To determine the ability of Treg-of-B cells to protect against colitis induced by colitogenic T cells, CD4^+^CD45RB^hi^ colitogenic T cells were adoptively co-transferred into SCID mice, with either PBS (control), Treg-of-B cells or CD4^+^CD45RB^lo^ cells. A decrease in body weight was used as a measurement of disease progression. After eight weeks, the mice that were given Treg-of-B or CD4^+^CD45RB^lo^ cells had similar body weight. However, the body weight of mice that were given PBS as a control had decreased significantly (Fig. 2A). Colitis symptoms were also presented in mice given colitogenic T cells alone, but not in mice that were also co-injected with Treg-of-B cells or CD4^+^CD45RB^lo^ cells (Fig. 2D). The histological analysis of the colonic tissue showed the presence of disease, characterized by an infiltration of leukocytes, depletion of goblet cells, and epithelial hyperplasia. In contrast, mice that were given Treg-of-B cells had mild colitis features, and mice treated with CD4^+^CD45RB^lo^ cells had sporadic colitis features (Fig. 2B,C).

### Treg-of-B cells inhibited Th1/Th17 cytokine protected *in vivo*

**Figure 3:**
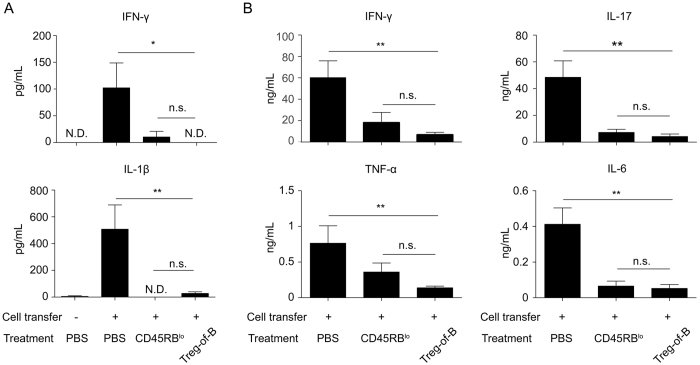
Treg-of-B cells inhibited Th1 and Th17 cytokine production in mice with colitis. Cytokine production in cells from the colon tissue cultures (**A**) and MLN cells (**B**) was measured by ELISA. The results are represented as the mean ± SEM. Significant differences were calculated using a one-way ANOVA. *P < 0.05; **P < 0.01; ***P < 0.001; not significant (NS) indicates P > 0.05 for select comparisons. There were 5 mice per group. The data are representative of two independent experiments.

To address whether the addition of Treg-of-B cells can suppress inflammatory cytokine production in the colons of mice adoptively transferred with colitogenic T cells, the levels of cytokines in cultured supernatant from colon explants and mesenteric lymph nodes (MLNs) were assessed. The levels of both IFN-γ and IL-1β in colon cultures significantly decreased in mice that had received either CD4^+^CD45RB^lo^ or Treg-of-B cells, and there were no significant differences between these groups (Fig. 3A). Co-transfer of either CD4^+^CD45RB^lo^ or Treg-of-B cells reduced the levels of IFN-γ, tumor necrosis factor (TNF)-α, IL-6, and IL-17 in the MLNs of mice with colitis. Therefore, Treg-of-B cells reduced Th1 and Th17 cytokine production to a level comparable to CD4^+^CD45RB^lo^ cells (Fig. 3B).

### IL-10-deficient Treg-of-B cells exerted the regulatory function

**Figure 4:**
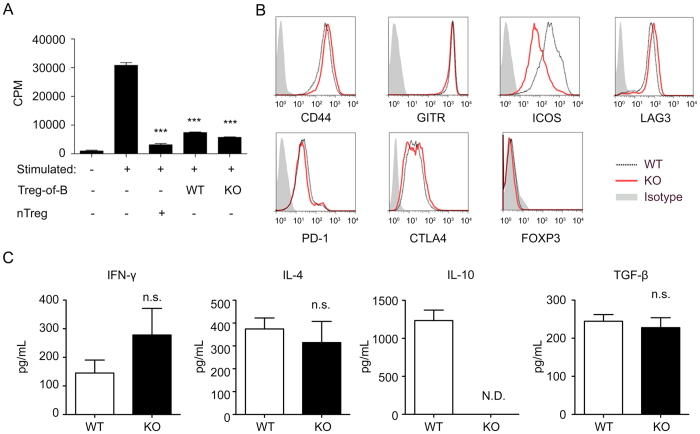
Treg-of-B cells mediated their regulatory function through an IL-10-independent mechanism. (**A**) The suppressive function of WT or IL-10 KO Treg-of-B cells was analyzed with nTreg cells as a suppressor control. (**B**) Representative histograms show the expression of each marker on gated CD4^+^CD25^+^ WT or KO Treg-of-B cells. (**C**) Cytokine levels of stimulated WT and KO Treg-of-B cells were analyzed by ELISA. *P < 0.05; **P < 0.01; ***P < 0.001; not significant (NS) indicates P > 0.05 for select comparisons. The results are shown as the mean ± SEM. Significant differences were calculated using Student’s *t*-test. The data are representative of three independent experiments.

Our recent study showed that IL-10-deficient B cells still induced Treg-of-B cells. We investigated whether IL-10 is essential for the regulatory function of Treg-of-B cells both *in vivo* and *in vitro*. Thus, we analyzed the suppressive ability of Treg-of-B cells using both BALB/c (WT) and *Il10*^−/−^ (IL-10 knockout, IL-10 KO) Treg-of-B cells. As shown in Fig. 4A, IL-10 deficiency had no significant effect on the suppressive function of Treg-of-B cells. To determine whether IL-10 KO Treg-of-B cells expressed additional suppressive molecules to compensate for the lack of IL-10, we compared the signatures of WT and IL-10 KO Treg-of-B cells. The majority of the Treg-associated markers expressed by WT and IL-10 KO Treg-of-B cells were the same. However, the expression of ICOS was lower in IL-10 KO Treg-of-B cells compared to that of WT Treg-of-B cells (Fig. 4B). Next, we examined the cytokine profiles of WT and IL-10 KO Treg-of-B cells. We found that that the IL-10 KO Treg-of-B cells produced slightly higher level of IFN-γ, yet similar amounts of IL-4 and TGF-β. As expected, IL-10 KO Treg-of-B cells did not produce IL-10 (Fig. 4C).

### IL-10 KO Treg-of-B cells protected against colitis

**Figure 5:**
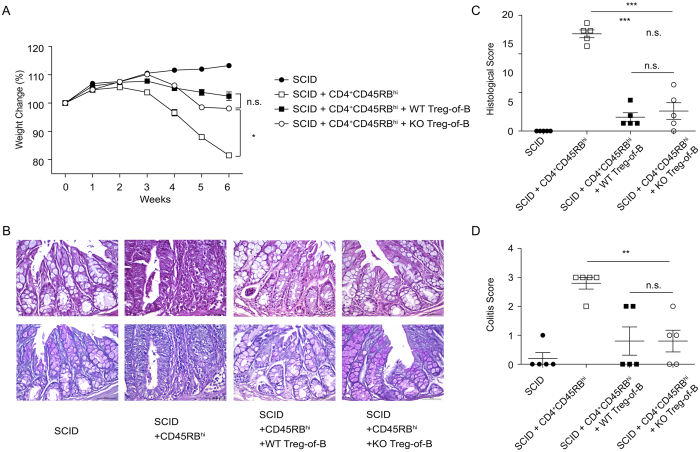
IL-10-deficient Treg-of-B cells suppressed the development of colitis. (**A**) Relative body weight changed over time, (**B**) histological sections of the colon (*top*, H&E staining; *bottom*, periodic acid-Schiff staining; scale bar, 50 μm) and (**C**) histological scores of the colons from mice that received CD4^+^CD45RB^hi^ T cells are shown. (**D**) Disease severity scores from mice with colitis were carried out six weeks after the transfer of CD4^+^CD45RB^hi^ T cells. The results are represented as the mean ± SEM. Significant differences were calculated using a one-way ANOVA. *P < 0.05; **P < 0.01; ***P < 0.001; not significant (NS) indicates P > 0.05 for select comparisons. The statistics are shown in the last time in (**A**). The data are representative of two independent experiments.

To assess whether IL-10 is necessary for Treg-of-B cells to protect against colitis, CD4^+^CD45RB^hi^ effector T cells were adoptively transferred into SCID mice with either WT or IL-10 KO Treg-of-B cells. After six weeks, mice that had received either WT or IL-10 KO Treg-of-B cells exhibited less weight loss (Fig. 5A), milder histopathology (Fig. 5B,C), and milder colitis features (Fig. 5D) compared with mice that only received colitogenic T cells. Also, IL-10 KO Treg-of-B cells protected against colitis to a similar extent as WT Treg-of-B cells.

**Figure 6:**
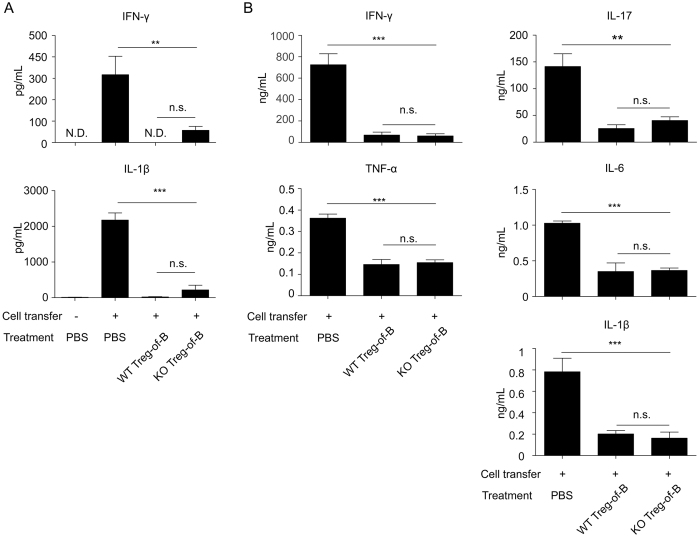
IL-10-deficient Treg-of-B cells inhibited Th1 and Th17 cytokine production in mice with colitis. Cytokine production in cells from the colon tissue cultures (**A**) and MLN cells (**B**) was measured by ELISA. The results are represented as the mean ± SEM. Significant differences were calculated using a one-way ANOVA. *P < 0.05; **P < 0.01; ***P < 0.001; not significant (NS) indicates P > 0.05 for select comparisons. There were 5 mice per group. The data are representative of two independent experiments.

We next examined the levels of inflammatory cytokines in culture supernatants from colon explants and MLNs. Compared with mice that received colitogenic T cells alone, mice that were co-transferred with either WT or IL-10 KO Treg-of-B cells had reduced levels of IFN-γ and IL-1β in the colon (Fig. 6A), and reduced amounts of IFN-γ, TNF-α, IL-17, IL-6, and IL-1β in the MLNs (Fig. 6B). Importantly, there was no difference between the mice that had received WT Treg-of-B cells and those that received IL-10 KO Treg-of-B cells.

## Discussion

In this study, we investigated the characteristics and regulatory function of Treg-of-B cells and examined whether IL-10 is necessary for the protective effects. Using an adoptive transfer colitis model in SCID mice, we found that Treg-of-B cells were able to successfully inhibit disease progression. We also examined whether IL-10 KO Treg-of-B cells retained the capability to suppress T cell proliferation and prevent colitis, both *in vivo* and *in vitro*. Overall, we investigated the function of Treg-of-B cells and found that these cells were different from those of IL-10-producing Tr1 cells.

B cells are important to maintain intestinal homeostasis and exert their function by secreting immunoglobulin and cytokines. In addition to their effector functions, regulatory B (Breg) cells modulate Treg cell development, proliferation, and survival^19^. The absence of B cells result in the severe and rapid onset of experimental autoimmune encephalomyelitis and coliti suggesting that there is a link between B cells and Treg cells^20^. Moreover, recent studies suggest that B cells can actively induce Treg cells. Orally administration of cholera toxin B conjugated antigen increase the frequency of antigen-specific Foxp3^+^ T but not in B cell-deficient mice^21^. IL-10-secreting B cells can induce Tr1 cells and thereby ameliorate T cell-mediated colitis^22^. In addition, the cell-cell interactions between antigen-specific B cells and naïve T cells creates a mature immunologic synapse and can induce the de novo generation of Treg cells^6^,^23^. Our present results also suggested that antigen-nonspecific Treg-of-B cells induced by conventional B-2 cells in the presence of anti-CD3 and anti-CD28 antibodies^9^ (Fig. 1).

The linage factor Foxp3 has long been known as an essential regulator of Treg cells for their function and development^24^. However, recent studies reported the existence of the Foxp3^−^ Treg subsets, including Tr1 and CD4^+^CD25^−^LAG3^+^ T cells^2^. Here, we found that Treg-of-B cells, induced by anti-CD3 and anti-CD28 antibodies, were also Foxp3^−^ suppressive T cells (Fig. 1A). We further analyzed the characteristics of Treg-of-B cells, the phenotype and the production of IL-10 were similar to those of nTreg and Tr1 cells^25^,^26^ (Fig. 1A,B).

The immunosuppressive function of Treg-of-B cells has been shown in many animal disease models. Reichardt et al. demonstrated that Treg-of-B cells potently suppress cutaneous hypersensitivity and ectopic allogeneic heart transplant rejection^6^. Our group has also demonstrated that Treg-of-B cells induced by splenic B-2 and Peyer’s patch B cells modulated airway inflammation in the murine model of allergic asthma^7^,^27^. The present study examined the function of Treg-of-B cells in a T cell-mediated colitis model. The co-transfer of Treg-of-B cells with CD4^+^CD45RB^hi^ effector T cells into SCID mice effectively inhibited the symptoms of colitis and reduced the production of Th1 and Th17 cytokines in the MLNs and colons (Figs 2 and 3). These protective effects are similar to those seen with the co-transfer of CD4^+^CD45RB^lo^ T cells, which is a Treg-enriched T cell population that has been previously shown to inhibit the onset of colitis^28^.

Many studies have shown that the mucosal production of IL-10 promote intestinal tolerance. IL-10 has been shown to suppress Th1 and Th17 responses in colitis^29^,^30^,^31^, and Tr1 cells induced by immature dendritic cells also require IL-10^32^. CD4^+^CD25^−^LAG3^+^ T cells protect mice form colitis in an IL-10-dependent manner^4^. We speculated that IL-10, which is highly secreted by Treg-of-B cells, might be responsible for the protective effects of Treg-of-B cells in colitis. However, our *in vitro* and *in vivo* data show that IL-10 deficiency did not affect the immune regulatory functions of Treg-of-B cells. IL-10 KO Treg-of-B cells successfully suppressed responder T cell proliferation (Fig. 4A); moreover, IL-10 KO Treg-of-B cells were able to attenuate chronic colitis induced by the colitogenic T cells (Fig. 5) and suppress the Th1 and Th17 response (Fig. 6). We hypothesized that IL-10 KO Treg-of-B cells might upregulate other immune modulator molecules to compensate for the loss of IL-10, but we did not find increased expression of any other regulatory molecules in this study (Fig. 4B,C).

It is unclear that IL-10 production was not necessary for Treg-of-B cells to protect against colitis in our study. Although IL-10 KO CD4^+^CD25^+^ Treg cells are less effective than WT cells, they can still prevent T cell-mediated colitis^33^. These suggest that Treg cells can inhibit colonic inflammation through other mechanisms other than the secretion of IL-10. On the other hand, our previous data showed that the suppressive capability of Treg-of-B2 cells reduced in the presence of a transwell insertion, suggested that Treg-of-B2 cell-mediated suppression required cell-cell contact^9^. These results suggest that surface molecules expressed on IL-10 KO Treg-of-B cells may play a role in the suppressive function.

Treg-of-B cells expressed several regulation-associated molecules, including CTLA-4, GITR, OX40, LAG3, and PD-1. These molecules in Treg cells can control the activation of antigen presenting cells and lead to the accumulation of Treg cells in the colon^34^,^35^,^36^,^37^,^38^. Our group also found that LAG3^+^ Treg-of-B cells induced by Peyer’s patch B cells could alleviate airway hypersensitivity^8^. Taken together, these data provide hints about how IL-10 KO Treg-of-B cells utilize other regulatory pathways to attenuate the severity of colitis.

We also found that Treg-of-B cells share a similar phenotype with Tr1 cells. Currently, there is no lineage-defining transcription factor or signature cellular surface markers for Tr1 cells. Their characterization is based on cytokine profile (IL-10^hi^ IL-4^−^ IFN-γ^lo^) and IL-10-dependent suppression mechanisms^26^,^39^. In vitro cultued, OVA-specific Tr1 cells prevent colitis through the IL-10 production^16^. Therefore, IL-10-independent regulatory mechanisms might provide a unique feature to distinguish Treg-of-B cells from Tr1 cells. Our group found that Treg-of-B cells did not express CD49b or CD103, both of which expressed in Tr1 cells^8^. In addition, IL-10 KO B2 cells still induced Treg-of-B cells^9^, whereas the induction of Tr1 cells requires IL-10^16^.

**Figure 7:**
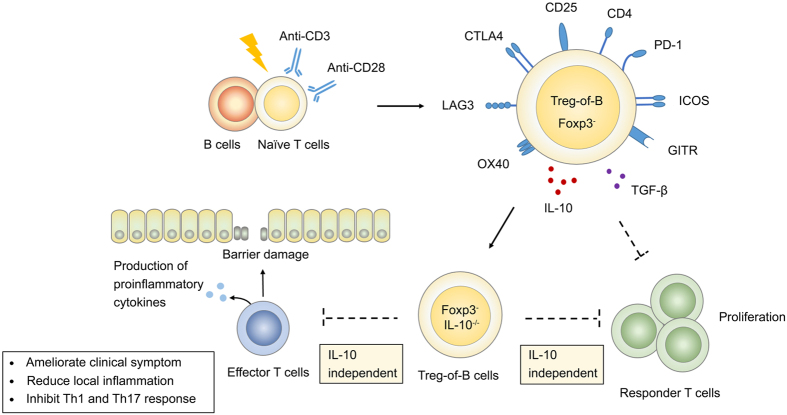
The schematic figure demonstrated the immunoregulatory function of Treg-of-B cells. B220^+^ splenic B cells could convert naïve T cells into Treg-of-B cells in the presence of anti-CD3 and anti-CD28 antibodies. Treg-of-B cells upregulated Treg cells associated molecules and secreted IL-10 and TGF-β, and inhibited the proliferation of responder T cells in an IL-10-independent manner. Adoptive transferring Treg-of-B cells could also ameliorate T cell mediated colitis and downregulated the Th1 and Th17 responses. Both of the immunomodulatory process could be through an IL-10-independent mechanism.

In conclusion, these findings shed new light on Treg-based therapies for experimental colitis. Treg-of-B cells inhibited colitis and suppressed Th1 and Th17 responses in an IL-10-independent manner. In addition, unlike the IL-10-dependent regulatory mechanisms of Tr1 cells, IL-10 is not necessary for Treg-of-B cell-mediated suppression (Fig. 7). Our study here is the first one to demonstrate the effectiveness of IL-10 deficient Treg-of-B cells *in vivo*. The generation of Treg-of-B cells *in vitro* might potentially be utilized as a new approach for IBD therapy. However, further studies are needed to understand the detailed immune modulatory mechanisms of Treg-of-B cells, distinguish them from other Treg subtypes and utilize Treg-of-B cells therapeutically in human IBD.

## Methods

### Mice

Female C.B17/Icr-*Prkdc*^*SCID*^/Crl (SCID) and BALB/c mice were from the National Laboratory Animal Center aged 6–8 weeks. BALB/c *Il10*^*−/−*^ (KO) mice were purchased from Jackson Laboratory. All mice were maintained in Laboratory Animal Center of the College of Medicine at National Taiwan University. All animal experiments were approved by the Institutional Animal Care and Use Committee at College of Medicine, National Taiwan University (license number 20120193), and performed in accordance with the approved guidelines.

### Cell preparation

The B220^+^ B cells were isolated from splenocytes of BALB/c mice. CD4^+^CD25^−^ T (T_naïve_) and CD4^+^CD25^+^ T (nTreg) cells were isolated from splenocytes of BALB/c and BALB/c *Il10*^*−/−*^ mice. Each cell population was purified by immunomagnetic positive or negative selection via BD IMag cell purification system (BD PharMingen, San Diego, CA, USA) according to the manufacturer’s instructions.

### *In vitro* generation of Treg-of-B cells

CD4^+^CD25^−^ T cells and B220^+^ B cells were isolated from BALB/c or *Il10*^*−/−*^ mice, and added into the culture at a 1:1 (B/T) ratio in the presence of 0.5 μg mL^−1^ anti-CD3 and anti-CD28 antibodies (Biolegend, San Diego, CA, USA). After 3 days, Treg-of-B cells were obtained after depleting the B220^+^ cells.

### Proliferation assay

In the suppressive assay, Treg-of-B cells (1 × 10^5^) were cultured with purified CD4^+^CD25^−^ T cells (1 × 10^5^) and irradiated splenocytes (3000 cGy) (1 × 10^5^) in presence of 2 μg mL^−1^ ConA mitogen for 72 hrs. The proliferation was assessed by [^3^H]-thymidine incorporation (1 μCi per well, PerkinElmer, Boston, Mass), was added after 72 hrs cultured and harvested for 16–18 hrs. Cells were analyzed by β-counter (Packard Instrument Co., Meriden, CT, USA) and presented in counts per minute (CPM).

### Cytokine analysis

To determine the levels of cytokines, CD4^+^CD25^−^ T cells, CD4^+^CD25^+^ T cells, and Treg-of-B cells (5 × 10^5^) were restimulated with the mitogen ConA and irradiated splenocytes (5 × 10^5^) for 48 hrs. MLN cells (1 × 10^6^) were stimulated with 2 μg mL^−1^ anti-CD3 antibody and cultured for 72 hrs.

To analyze the production of cytokines, an ELISA was performed using the DuoSet ELISA Development kit (R&D, Minneapolis, MN, USA) according to the manufacturer’s instructions. The following cytokines were assayed: IL-1β, IL-4, IL-6, IL-10, IL-17, TNF-α, IFN-γ, and TGF-β.

### Colonic tissue explant cultures

Two-centimeter sections from the middle part of the colon were excised, halved longitudinally, and washed three times with sterile cold PBS. The processed colon tissue was then cultured in 2 mL of complete RPMI 1640 medium. After 3 days, supernatants were collected.

### Flow cytometry and cell sorting

The staining procedures were performed according to the manufacturer’s instructions. To analyze cellular markers, the cells were stained with the following fluorescence-conjugated monoclonal antibodies: anti-CD4 (GK1.5), anti-CD25 (PC61.5), anti-LAG3 (C9B7W), anti-GITR (DTA-1), anti-CTLA4 (UC10-4B9), anti-OX40 (6X-86), anti-ICOS (15F9), anti-CD44 (IM7), anti-PD-1 (J43), and anti-Foxp3 (FJK-16s) all of which were purchased from eBioscience (San Diego, CA, USA). Flow cytometry was performed using a BD FACSCalibur (BD Biosciences, San Jose, CA, USA).

Cells were sorted on FACSAria (BD Bioscience, San Jose, CA) through the service provided by the Flow Cytometric Analyzing and Sorting Core (the First Core Laboratory, National Taiwan University College of Medicine).

### Induction of colitis and adoptive transfer of Treg-of-B cells

SCID mice were intraperitoneal (i.p.) injected with CD4^+^CD45RB^hi^ effector T cells (5 × 10^5^ per mouse) from BALB/c mice. Subsequently, mice were i.p. injected with CD4^+^CD45RB^lo^ T cells (2.5 × 10^5^ per mouse), Treg-of-B cells (3 × 10^6^ per mouse) or PBS. The body weight of each mouse was recorded weekly to assess disease severity.

### Colitis scoring and histological assessment of tissue sections

The criteria for the colitis disease score has been described elsewhere^40^. The mice were clinically evaluated prior to their sacrifice. Colitis disease score was scored as follows: 0, normal; 1, soft or loose stools; 2, diarrhea; 3, blood in stools or prolapsed rectum.

The colon sections were fixed with 10% formalin and embedded in paraffin. The sections were then stained with either hematoxylin and eosin or periodic acid–Schiff stain. The severity of inflammation in colon section was evaluated by a veterinary pathologist. Histology was scored as follows: 1) severity of inflammation: 0, none; 1, minimal, multifocal infiltration of lymphocytes in the mucosal layer; 2, slight, multifocal infiltration of lymphocytes in the mucosal layer; 3, moderate, diffuse infiltration of lymphocytes and neutrophils in the mucosal and submucosal layers; 4, severe, diffuse infiltration of lymphocytes and neutrophils in all transmural layers; 2) hyperplasia: 0, normal (≤100%); 1, slight (101–150%); 2, moderate (151–200%); 3, severe (>200%); 3) amount of mucus: 0, normal; 1, Slight decrease of mucus; 2, moderate decrease of mucus; 3, Severe depletion of mucus; 4) degeneration of crypt and mucosa: 0, none; 1, slight (<5 per section); 2, moderate (5–10 per section); 3, severe (>10 per section); 5) erosion of epithelial and mucosa: 0, none; 1, slight, multifocal; 2, moderate, multifocal; 3, severe, diffuse.

### Statistical analysis

The statistical analyses were performed using GraphPad Prism 5 software. Significant differences were determined using the unpaired Student’s *t*-test or one-way Analysis of Variance (ANOVA). A value of P < 0.05 was considered to be statistically significant. All of the data were expressed as the mean ± SEM.

## Additional Information

**How to cite this article**: Shao, T.-Y. *et al*. Novel Foxp3^−^ IL-10^−^ Regulatory T-cells Induced by B-Cells Alleviate Intestinal Inflammation in Vivo. *Sci. Rep*. **6**, 32415; doi: 10.1038/srep32415 (2016).
